# Study on painless gastroscopy and POCD of smoking patients under general anesthesia

**DOI:** 10.1002/ibra.12041

**Published:** 2022-05-25

**Authors:** Wan‐Qiu Yu, Yu‐Hang Zhu, Xin‐Xin Yang, Tao‐Wu Gong, Zhen‐He Yu, Zhen‐Yu Wu, Liang Dong, Zhao‐Qiong Zhu

**Affiliations:** ^1^ Department of Anesthesiology Affiliated Hospital of Zunyi Medical University Zunyi Guizhou China; ^2^ Liuzhou People's Hospital Affiliated to Guangxi Medical University Liuzhou China

**Keywords:** anesthesia, painless gastroscopy, postoperative cognitive dysfunction, smoking

## Abstract

The number of smoking patients receiving anesthesia and surgical treatment is increasing day by day. It will be useful for medical advancement to explore whether smoking is an independent risk factor for postoperative cognitive impairment. A double‐blind, parallel, and controlled study was conducted on 112 patients who fulfilled the criteria for inclusion in this study and planned to undergo painless gastroscopy under general anesthesia. The baseline mini‐mental state examination (MMSE) scores and basic information were collected. The changes in the MMSE scores after waking up and 3 days after anesthesia were observed, and the adverse events (respiratory adverse reactions, circulatory fluctuations, and adverse reactions, drug use, etc.) were analyzed by logistic regression. The baseline level of each group is consistent, which is worth studying. The MMSE score of the smoking group after anesthesia was significantly different from that of the control group (*p* < 0.05), but there was no significant difference between the two groups 3 days after anesthesia. Among them, the differences in adverse events between the two groups were in terms of hiccup, postoperative cough, and SpO_2_ < 90% (*p* < 0.05). Regression analysis indicates that smoking after anesthesia leads to the occurrence of postoperative cough. Smoking is probably an independent risk factor for post‐operative cognitive dysfunction (POCD) in early postoperative patients.

## Graphical abstract

The purpose of this article was to explore whether smoking is an independent risk factor for postoperative cognitive impairment. A double‐blind, parallel, and controlled study was conducted on 112 patients who met the standard and planned to undergo painless gastroscopy under general anesthesia. The changes in mini‐mental state examination (MMSE) scores after waking up and 3 days after anesthesia and the adverse events were observed. The study found that in patients with chronic smoking after undergoing general anesthesia, the MMSE score would decline after they awoke, it indicated that smoking patients with traumatic stimulus in the absence of general anesthesia would display transient mild cognitive function, smoking is probably an independent risk factor for post‐operative cognitive dysfunction in early postoperative patients.

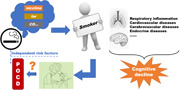

## 1 INTRODUCTION

Smoking has become a serious public health problem worldwide. The respiratory, circulatory, and endocrine systems of long‐term smokers tend to be damaged to different degrees. Smoking stimulates alveolar macrophages and polynuclear leukocytosis, and mediates chronic nonspecific inflammation, which even triggers systemic chronic inflammation.^1^, ^2^ At the same time, smoking can also induce atherosclerosis through oxidative stress in the vascular endothelium, thereby inducing cardiovascular and cerebrovascular diseases.^3^ According to the statistics, smoking accounts for 30% of the 230 million surgical patients every year.^4^ Whether smoking becomes an independent risk factor for postoperative cognitive dysfunction after general anesthesia remains unknown.

Previous studies have shown that postoperative cognitive dysfunction is mainly associated with advanced age, cardiovascular and cerebrovascular diseases, mental diseases, surgical trauma, and other factors. Nicotine present in tobacco exerts effects on the central nervous system and related neurotransmitters, and has a certain relationship with changes in cognitive levels.^5^ Research by Wang et. al pointed out that preoperative smoking history is associated with a reduced risk of early postoperative cognitive dysfunction in elderly patients after noncardiac surgery, which may be related to the activation of the nicotine‐mediated cholinergic anti‐inflammatory pathway and central nervous system inflammation or inhibition of inflammatory factors (TNF‐α, IL‐1β, and IL‐6).^6^ This study seems to be inconsistent with our traditional belief that smoking may exert chronic central system effects and that cognitive dysfunction may occur after anesthesia surgery, which makes it all the more necessary to continue such systematic research.

Painless gastroscopy is a short, atraumatic medical diagnosis and can be performed under general anesthesia. Therefore, in this study, to reduce the interference of multiple factors such as operation duration and trauma, we selected smoking patients undergoing painless gastroscopy under general anesthesia as research participants and aimed to determine whether smoking is an independent risk factor for post‐operative cognitive dysfunction (POCD) by carrying out a prospective double‐blind controlled study.

## 2 MATERIALS AND METHODS

The research protocol was approved by the Ethics Committee of the Affiliated Hospital of Zunyi Medical University (Approval No: KLLY‐2019‐091). The patient and his/her family members were aware of the research protocol and voluntarily signed the informed consent form. In this study, inclusion and exclusion criteria were strictly controlled. A total of 120 outpatients undergoing painless gastroscopy in our hospital were included as research participants from May 2020 to August 2020. Eight patients were excluded, and finally, a total of 112 patients were followed up.

### 2.1 Sample size calculation

Using the PASS11.0 system, changes in the postoperative mini‐mental state examination (MMSE) scale were used as the main evaluation index. After preexperimental observations (12 patients in the control group and 15 patients in the experimental group), the mean values of the two groups were 26.3 ± 2.2 and 24.1 ± 3.4, the setting efficacy was 0.8, *α* = 0.05, and the bilaterally calculated sample size was 100. Based on the expulsion rate of 20%, the total sample size was set as 120 cases, 60 cases in each group.

### 2.2 Patients

Patients who were scheduled for gastroscopy with propofol sedation, with examination time between 10 and 30 min, patients between 18 and 65 years of age, patients who were American Society of Anesthesiologists (ASA) classes Ⅰ–Ⅱ, and with body mass index between 18 and 28 kg/m^2^, and patients who provided written informed consent were invited to participate in the study. Patients were excluded if they were aged <18 years or >65 years, were ASA class III‐V, had severe trauma or had undergone major surgery in the past, had hypertension (systolic blood pressure ≥ 180 mmHg in the supine position during the screening phase and/or diastolic blood pressure ≥ 110 mmHg) and hypotension (blood pressure < 90/60 mmHg) with unsatisfactory control of blood pressure after antihypertensive therapy, had severe cardiovascular history, had myocardial infarction within the last 6 months, had psychiatric disorders and cognitive dysfunction, or did not have the ability to cooperate. During the trial, they withdraw their informed consent, have clinical adverse events or other medical conditions that are not suitable for continuing the trial, and the examination time >30 min. Patients for whom follow‐up failed, patients in whom unexpected events unrelated to the trial occurred, patients who required general anesthesia again in a short time, and patients who did not cooperate during the study were not included in the final statistical analysis.

A total of 120 patients who were scheduled to undergo painless gastroscopy in our outpatient department were selected according to the inclusion and exclusion criteria and divided into two groups according to whether or not the patients smoked: the smoking group (*n* = 60) and the control group (*n* = 60). None of the patients had any other control variables, except for smoking history, and the observation indexes were the same, without additional intervention.

### 2.3 Procedure

After the patients signed the informed consent form, the basic information was collected: initials, outpatient number, demographic information, smoking history (smoking years and daily smoking volume), current medical history or reasons for this examination, previous medical history and medication history, surgical history, allergy history, general situation, ASA grade and improved Markov score, MMES score, and vital signs baseline (heart rate [HR], non‐invasive blood pressure [NIBP], respiration rate [RR], SpO_2_). It was ensured that the patients fulfilled the inclusion criteria of the study.

Patients voluntarily signed the consent form for endoscopy and anesthesia. The time of fasting and drinking was determined, and the MMSE score and MOAA/S baseline were obtained in the waiting room. 30 minutes before the examination, the patient was required to remove gastric mucus by oral administration of streptavidin particles. Anesthesia induction and maintenance were performed under routine clinical anesthesia without additional intervention in this study. In the gastroscopy room, the patient was placed in the left lateral decubitus position, and HR, NIBP, RR, SpO_2_, and electrocardiogram were recorded.

The patient received oxygen through a mask (4 L/min), and 50 μg of fentanyl was injected intravenously in advance. After the administration of fentanyl, a 2.0 mg/kg propofol injection was slowly administered intravenously. After intravenous injection of drugs, the respiratory frequency and SpO_2_ changes of the patients were closely monitored. Gastroscopy could be started once the patient's breathing slowed down and stabilized, the eyelash reflex disappeared, the muscles of the whole body were relaxed, and the MOAA/S score was ≤2 points. If the examination time was prolonged and the stimulation of operation is strong, 0.5 mg/kg propofol was injected intravenously every time according to the patient's physical signs such as breathing amplitude, heart rate, physical activity, and the MOAA/S score. During the study, if the patient's systolic blood pressure was ≤80 mmHg or the systolic blood pressure dropped by more than 30% of the basic value, 9 mg of ephedrine was injected intravenously to increase hypertension. If the patient's heart rate was lower than 50 times, intravenous administration of atropine 0.3 mg induced an increase in the heart rate. If the SpO_2_ of the patient was less than 90%, the oxygen inhalation flow was increased or high‐concentration oxygen was provided through a mask, and if necessary, the inlet pharyngeal airway was placed. After the examination, the patient was sent to the anesthesia recovery room with oxygen inhalation (2 L/min) through a mask. After the patient fully awakened, the anesthesiologist evaluated the patient. If the Aldrete score was not less than 9 points, the patient could be accompanied to leave the hospital.

### 2.4 Measurements

This study was divided into four periods: screening period, examination period, PACU observation period, and postoperative follow‐up period. The data recorded in each period are different. The data recorded by the filter are as mentioned above. The data recorded in the examination period are as follows: patient's vital signs before anesthesia induction and MOAA/S score baseline, anesthesia induction time, and drug dose. HR and NIBP of the patient were recorded every 3 min after the start of anesthesia induction, RR and SpO_2_ of the patient were recorded every 1 min until the end of the operation, and the MOAA/S score was determined once every 1 min after anesthesia induction until the patient fully awakened (the MOAA/S score reached 5 points three consecutive times); time variables (examination start and end time), drug addition times, and adverse reactions were also noted. The data recorded during the PACU observation period were as follows: HR, NIBP, RR, and SpO_2_ were recorded once every 5 min after the operation, and MOAA/S scoring was conducted once every 1 min until the patient fully awakened and his or her vital signs were stable. Awakening time and the time when Aldrete >9 points, adverse reactions and medications during the experiment, MMSE score of patients with Aldrete >9 points for three consecutive times. The patients were followed up by telephone 72 h after discharge from the hospital. The data recorded were as follows: MMSE score and adverse reactions of the patients. A satisfaction questionnaire was also filled out by the patients.

The main observation indexes of this study included the MMSE score at each time point and an analysis of smoking status. Secondary observation indexes included adverse respiratory reactions (respiratory inhibition, manual ventilation with mandible support required, auxiliary ventilation required, cough during operation, and cough after operation), circulatory system fluctuation and adverse reactions, drug use, and incidence of other adverse reactions.

### 2.5 Data analysis

SPSS18.0 statistical software was used for statistical analysis in this study. The data with a normal distribution were expressed as mean and standard deviation. An independent‐sample *t* test was used for comparison between groups. Data with nonnormal distributions were expressed as medians (interquartile range) using a nonparametric rank‐sum test. Enumeration data were expressed as the actual number and percentage of cases. The *χ* ^2^ test, the continuous correction chi‐square test, or the Fisher exact probability method was used between groups. *p* < 0.05 indicated a statistically significant difference.

The *Z*‐score was calculated according to the measured MMSE scores before anesthesia, after anesthesia, and 3 days after recovery from anesthesia. The calculated values before anesthesia and after recovery from anesthesia, before anesthesia, and 3 days after recovery from anesthesia were calculated. The postoperative test score was lower than the score recorded before surgery. If the *Z* score was ≥1.96, the patient was classified as showing abnormal cognition and logistic regression analysis was then performed.

## 3 RESULTS

**Figure 1:**
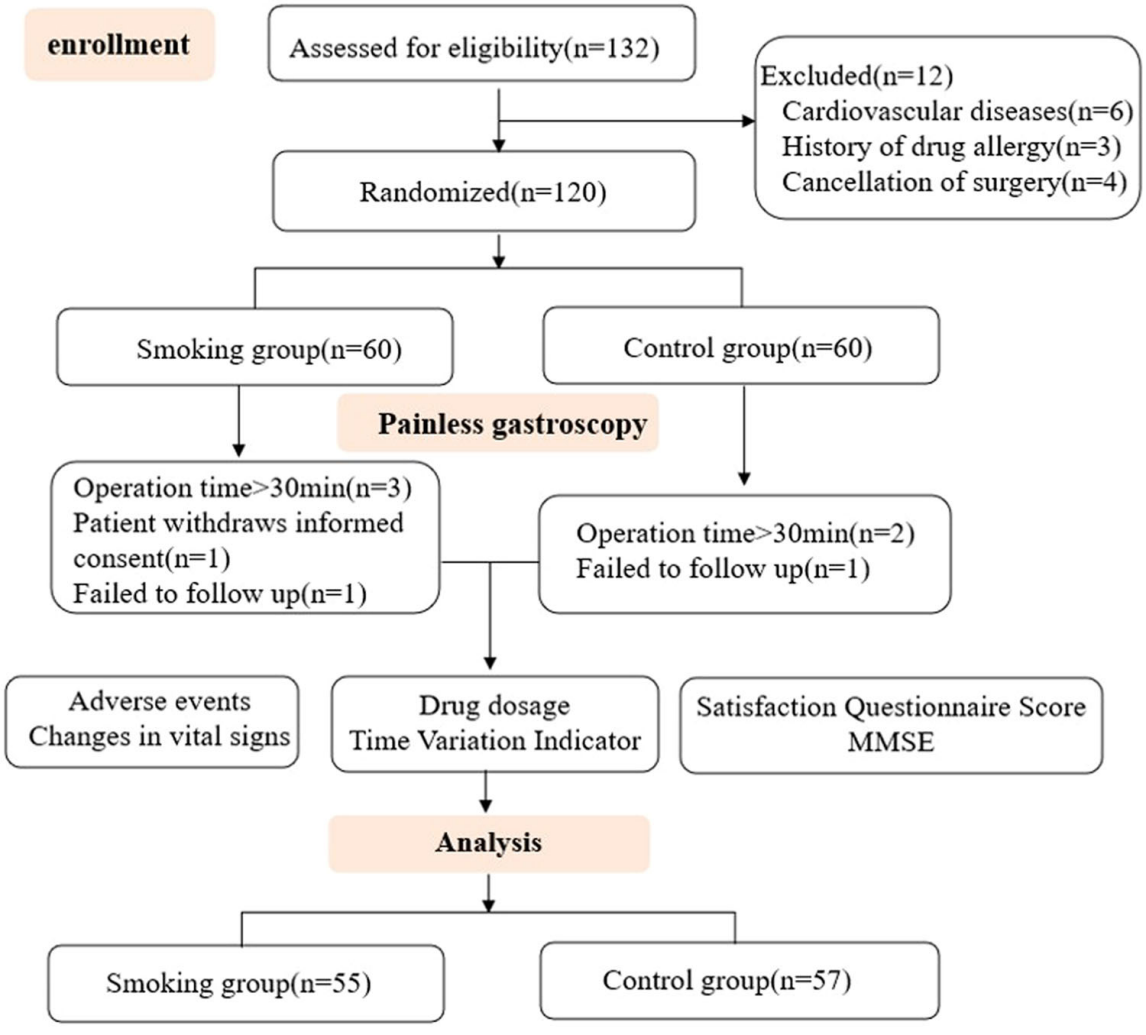
Flow chart of the study. [Color figure can be viewed at wileyonlinelibrary.com]

A total of 120 patients undergoing painless gastroscopy were included in this study. In the smoking group, after the examination began, three patients were excluded because the examination time was more than 30 min, one patient requested to withdraw from the study midway, and one patient dropped out due to follow‐up failure. In the control group, two patients whose examination time was more than 30 min were excluded, and one patient dropped out due to follow‐up failure. Finally, 55 patients in the smoking group and 57 patients in the control group completed the gastroscopy and follow‐up and were included in the statistical analysis (Figure 1). There was no significant difference in demographic characteristics such as age, height, weight, and body mass index between the two groups (*p* > 0.05). There were no significant differences between groups in physical examination, modified Markov score, ASA classification comparison, allergy history, previous medical history, and allergy history (*p* > 0.05). None of the patients had a history of allergy to opioids, propofol, Masini fluoride, naloxone, and other drugs and their drug components. This indicated that the two groups of patients were comparable (Table 1).

**Table 1:** Characteristics of the patients (*n* = 112).

	Smoking group (*n* = 55)	Control group (*n* = 57)	*p* value
Age (years), mean ± SD	46.53 ± 9.33	42.25 ± 10.22	0.207
Height (cm), mean ± SD	166.51 ± 5.62	159.44 ± 5.89	0.967
Weight (kg), mean ± SD	63.00 ± 8.02	56.62 ± 7.49	0.201
BMI (kg/m^2^), mean ± SD	22.79 ± 2.68	22.20 ± 2.81	0.982
Physical examination			
Normal	55	57	/
Abnormal	0	0	
Unchecked	0	0	
ASA, *n* (%)			
Ⅰ	5 (9.10）	14 (24.60)	0.054
Ⅱ	50 (90.90）	43 (75.40)	
Ⅲ	0 (0)	0 (0)	
Ⅳ	0 (0)	0 (0)	
Modified markov score, *n* (%)			
Ⅰ	40 (72.70)	40（70.20)	0.765
Ⅱ	15 (27.30)	17（29.80)	
Ⅲ	0 (0)	0 (0)	
Ⅳ	0 (0)	0 (0)	
Previous medical history, *n* (%)			
Positive	24 (43.60)	30 (52.60)	0.341
Negative	31 (56.40)	27 (47.40)	
Operation history, *n* (%)			
Positive	13 (23.64)	17 (29.80)	0.460
Negative	42 (76.36)	40 (70.20)	
Allergic history, *n* (%)			
Positive	0 (0)	3 (5.30)	0.255
Negative	55 (100)	54 (94.70)	
MMSE, mean ± SD	25.64 ± 3.82	26.71 ± 3.12	0.128

Abbreviations: ASA, American Society of Anesthesiologists, BMI, body mass index; MMSE, mini‐mental state examination.

### 3.1 Analysis of smoking history data

A total of 55 patients in the smoking group were included in this study. They all smoked more than 10 cigarettes per day, and the distribution of smoking years was as follows (Table 2).

**Table 2:** Smoking status of patients in the smoking group (*n* = 55).

	*n*	%
0–10 years	3	5.50
10–20 years	26	47.30
20–30 years	0	0
30–40 years	22	40.00
>40 years	4	7.30
Total	55	100.00

### 3.2 Baseline of vital signs

The baseline vital sign indicators in the screening period in the smoking group and the control group were within the normal range, and there was no significant difference between the groups (*p* > 0.05). The patients of the two groups were comparable (Table 3).

**Table 3:** The baseline of vital signs in the screening period (*n* = 112).

	Smoking group (*n* = 5)	Control group (*n* = 57)	*p* value
SBP (mmHg)	120.25 ± 12.94	123.86 ± 12.15	0.678
DBP (mmHg)	77.09 ± 10.38	78.19 ± 10.15	0.748
MAP (mmHg)	91.56 ± 10.65	93.42 ± 10.08	0.610
HR (bpm)	73.45 ± 10.67	80.63 ± 16.14	0.257
SpO_2_ (%)	96.89 ± 1.36	96.89 ± 1.36	0.514
RR (bpm)	17.93 ± 3.36	17.49 ± 2.90	0.299

Abbreviations: DBP, diastolic blood pressure; MAP, mean artery pressure; SBP, systolic blood pressure.

### 3.3 Adverse reactions

The number of patients with adverse reactions after induction of anesthesia was 34 (30.4%) in the smoking group and 32 (28.6%) in the control group, with no significant difference between the two groups (*p* > 0.05). All patients successfully underwent painless gastroscopy, and no serious adverse reactions occurred (Table 4).

**Table 4:** Adverse reactions (n = 112)

	Smoking group (*n* = 55)	Control group (*n* = 57)	*p* value
Adverse reaction, *n* (%)			
Positive	34 (61.8)	32 (56.1)	0.541
Negative	21 (38.2)	25 (43.9)	
Serious adverse reaction, *n* (%)			
Positive	0 (0)	0 (0)	/
Negative	55 (100)	57 (100)	

**Figure 2:**
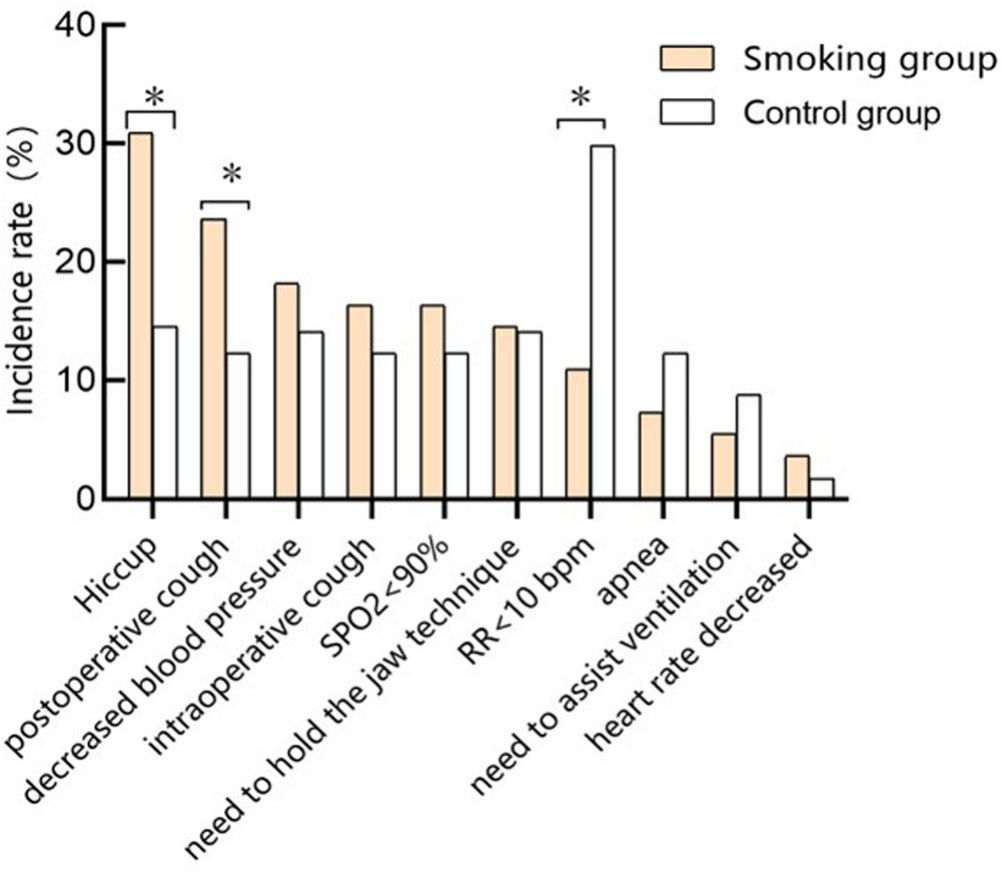
Comparison of the incidence of adverse reactions between the two groups. ^*^Comparison with the control group *p* < 0.05. [Color figure can be viewed at wileyonlinelibrary.com]

In the smoking group, the adverse reactions included hiccup (30.91%), postoperative cough (23.64%), decreased blood pressure (18.18%), intraoperative cough (16.36%), SpO_2_ < 90% (16.36%), the need for chin rest (14.55%), RR < 10 bpm(10.91%), apnea (7.27%), the need for auxiliary ventilation (5.45%), and HR slowdown (3.64%). In the control group, the adverse reactions included high RR < 10 bpm(29.82%), hiccup (14.55%), decreased blood pressure (14.04%), need for chin‐supporting manipulation (14.04%), postoperative cough (12.28%), intraoperative cough (12.28%), SpO_2_ < 90%(12.28%), apnea (12.28%), need of auxiliary ventilation (8.77%), and HR slowdown (1.75%) (Figure 2).

### 3.4 MMSE scores at different time points

Compared with the control group, the scores of the smoking group in MMSE after anesthesia were lower (*p* < 0.05), and there was no significant difference between the two groups before anesthesia and 3 days after anesthesia (Table 5).

**Table 5:** MMSE scores at different time points in the two groups (*n* = 112).

	Smoking group (*n* = 55)	Control group (*n* = 57)	*p* value
Before anesthesia	25.64 ± 3.82	26.71 ± 3.12	0.128
Awakening from anesthesia	23.37 ± 3.66^a^	25.83 ± 2.94	0.046
Three days after anesthesia	26.23 ± 4.12	27.27 ± 2.45	0.136

a Comparison with the control group *p* < 0.05.

### 3.5 Multivariate regression analysis

In univariate analysis, the statistically significant variables are displayed after the multicollinearity test, and no variables are excluded from the subsequent logistic regression analysis. Therefore, variables according to adverse reactions were included in the multivariate analysis. In multivariate analysis, MMSE scores of the cognitive state after anesthesia and 3 days after anesthesia were included (Table 6).

**Table 6:** Analysis of related factors of POCD.

Factors	Univariate analysis, *p* value	Multiple logistic regression analysis after anesthesia		Logistic regression analysis of 3 days after anesthesia	
		(95% CI)	*p* value	(95% CI)	*p* value
Smoking history	0.024	2.551 (1.843–2.643)	0.031	1.785 (1.334–1.860)	0.043
Hiccup	0.017	1.455 (1.018–1.672)	0.045	0.982 (0.945–1.023)	0.227
Postoperative cough	0.045	1.017 (0.961–1.121)	0.108	1.013 (0.971–1.087)	0.312
RR < 10 bpm	0.038	1.120 (0.949–1.189)	0.305	1.047 (0.957–1.059)	0.482

Abbreviations: CI, confidence interval, PCOD, post‐operative cognitive dysfunction.

## 4 DISCUSSION

Those who smoked more than one cigarette a day for 6 months are defined as long‐term smokers by the World Health Organization (WHO). Smoking is a serious public health problem recognized worldwide. Long‐term smoking may be one of the main factors leading to cardiovascular and cerebrovascular diseases. The incidence of coronary heart disease, hypertension, and other diseases in smoking patients is two to five times that of the general population, and the degree of harm increases with the increase in smoking years and the daily smoking amount.^7^ There are more than 4800 pharmacological active substances in cigarettes; the body will undergo a variety of pathophysiological changes if the person has been smoking for a long time. Many smokers take up the habit in adolescence, and the longer tobacco remains in the central nervous system, the more persistent the pathophysiological changes are likely to be. In particular, long‐term exposure to nicotine in adolescence may have a long‐term effect on cognitive function, which is related to the reduction of presynaptic mGluR 2 protein and excitatory synaptic function in the prefrontal cortex.^8^ The study of Suvi P. Rovio et al. showed that long‐term exposure to parents' second‐hand smoke in childhood may also lead to cognitive decline in adulthood.^9^ This suggests that long‐term exposure to nicotine or second‐hand nicotine may have detrimental effects on cognitive function.

With medical advancements and improved health care awareness, increasingly more people can receive optimal medical treatment under general anesthesia. This raises the question of whether general anesthesia increases the incidence of postoperative cognitive dysfunction in smoking patients. Is smoking an independent risk factor for postoperative cognitive dysfunction? At present, there are multiple hypotheses about the pathogenesis of postoperative cognitive dysfunction, including the influence of cholinergic receptors and central inflammation, which may lead to the occurrence of POCD.^10^, ^11^ Nicotine acts on endothelial nicotinic acetylcholine receptors (nAChRs) to activate endothelial cells and enhance pathological angiogenesis, in which α7nAChR plays a key role in mediating the effects of nicotine on endothelial cells.^12^ Studies have shown that inhibition of HMGB1‐NF‐κB by the α‐7nAChR signal can promote the occurrence of POCD.^13^ These results suggest that cholinergic receptor regulation plays a key role in POCD. This study found that patients with chronic smoking after receiving general anesthesia, the MMSE score will drop after awakening, appeared the performance of mild cognitive dysfunction, and there is a statistical difference compared with the control group, indicating that smoking patients in non‐invasive operations such as gastroscopy after general anesthesia will appear transient mild decline in cognitive function. Is there a relationship between this and central cholinergic changes? This can be further verified by animal models in future studies. The regression analysis found that smoking was an independent risk factor, which further explained the relationship between smoking and early POCD.

In this study, it was also found that postoperative cough expectoration and decreased oxygen saturation may also affect the occurrence of POCD in the early postoperative period. The airway of chronic smoking patients is stimulated for a long time, and nicotine, hydrogen cyanide, nitrite, and other substances in smoke are deposited in various parts of the lung, which leads to lung inflammation by inducing the activation of neutrophils and macrophages, and the inflammatory process may be an important factor in the progression of lung injury and lung function impairment.^14^ Studies have shown that adverse reactions such as choking, apnea, bronchospasm, and hypoxemia during induction of general anesthesia are related to smoking history.^15^ The occurrence and migration of lung inflammation can lead to central nervous system inflammation. In recent years, chronic lung inflammation was found to occur in some infected persons due to COVID‐19. Studies have shown that mast cells stimulated by long‐term COVID syndrome after novel coronavirus infection release neuroinflammation, which activates microglia and leads to hypothalamic inflammation.^16^ Smoking is likely to stimulate mast cells in the oral cavity and lung, resulting in chronic periodontitis and pneumonia, and then migrate and cause chronic inflammation.^17^ Further evolution of various types of chronic inflammation leads to the occurrence of central chronic insidious inflammation. POCD is affected by stimulation such as anesthesia and surgery or hypoxia in the brain.^18^, ^19^, ^20^ Previous studies of our research group also found that activation of microglia caused by inflammasomes may lead to POCD in rats.^21^ These factors may also represent another major pathway of POCD induced by smoking.

## 5 CONCLUSION

In conclusion, this study shows that smoking is likely to be an independent risk factor for POCD in early postoperative patients, and further research is needed to determine whether the mechanism is related to the action of cholinergic receptors and the occurrence of neuroinflammation.

## AUTHOR CONTRIBUTIONS

Wan‐Qiu Yu was responsible for experimental design, data collection, analysis, and article writing, Yu‐Hang Zhu was responsible for experimental guidance and quality control, Zhao‐Qiong Zhu and Liang Dong were responsible for experimental guidance and problem discussion, Xin‐Xin Yang and Zhen‐He Yu were responsible for experimental data collection, Tao‐Wu Gong was responsible for clinical safety assurance, and Zhen‐Yu Wu was responsible for data analysis.

## CONFLICTS OF INTEREST

Zhao‐Qiong Zhu and Yu‐Hang Zhu are reviewers for Ibrain but are not involved in the peer review process of this manuscript. Other authors declare no conflict of interest.

## ETHICS STATEMENT

This study followed the declaration of Helsinki and was approved by the ethics committee of the Affiliated Hospital of Zunyi Medical University (Approval No: KLLY‐2019‐091). During the study, the subjects agreed to participate and signed the informed consent form.

## TRANSPARENCY STATEMENT

All the authors affirm that this manuscript is an honest, accurate, and transparent account of the study being reported; that no important aspects of the study have been omitted; and that any discrepancies from the study as planned (and, if relevant, registered) have been explained.

## Data Availability

The authors confirm that the data supporting the findings of this study are available within the article and its supplementary materials.
